# Cross-platform analysis of public responses to the 2019 Ridgecrest earthquake sequence on Twitter and Reddit

**DOI:** 10.1038/s41598-022-05359-9

**Published:** 2022-01-31

**Authors:** Tao Ruan, Qingkai Kong, Sara K. McBride, Amatullah Sethjiwala, Qin Lv

**Affiliations:** 1 Department of Computer Science, University of Colorado Boulder, 430 UCB, Boulder, CO 80309 USA; 2 Lawrence Livermore National Laboratory, 7000 East Ave, Livermore, CA 94550 USA; 3 U.S. Geological Survey, 345 Middlefield Road, MS 977, Menlo Park, CA 94025 USA

**Keywords:** Environmental social sciences, Natural hazards

## Abstract

Online social networks (OSNs) have become a powerful tool to study collective human responses to extreme events such as earthquakes. Most previous research concentrated on a single platform and utilized users’ behaviors on a single platform to study people’s general responses. In this study, we explore the characteristics of people’s behaviors on different OSNs and conduct a cross-platform analysis of public responses to earthquakes. Our findings support the Uses and Gratification theory that users on Reddit and Twitter are engaging with platforms that they may feel best reflect their sense of self. Using the 2019 Ridgecrest earthquakes as our study cases, we collected 510,579 tweets and 45,770 Reddit posts (including 1437 submissions and 44,333 comments) to answer the following research questions: (1) What were the similarities and differences between public responses on Twitter and Reddit? (2) Considering the different mechanisms of Twitter and Reddit, what unique information of public responses can we learn from Reddit as compared with Twitter? By answering these research questions, we aim to bridge the gap of cross-platform public responses research towards natural hazards. Our study evinces that the users on the two different platforms have both different topics of interest and different sentiments towards the same earthquake, which indicates the necessity of investigating cross-platform OSNs to reveal a more comprehensive picture of people’s general public responses towards certain disasters. Our analysis also finds that r/conspiracy subreddit is one of the major venues where people discuss the 2019 Ridgecrest earthquakes on Reddit and different misinformation/conspiracies spread on Twitter and Reddit platforms (e.g., “Big one is coming” on Twitter and “Nuclear test” on Reddit).

## Introduction

Online social networks (OSNs) have become an essential component of people’s everyday life and these different platforms serve as important hubs for public expression and interactions. Meanwhile, although most people use OSNs merely as a way of recording daily life, the potential insight behind the social media data goes far beyond. Previous studies have utilized data collected from OSNs to analyze public responses to extreme events including natural hazards or major social events^1–3^. While most of these existing studies characterize one single OSN in the context of specific events, our study explores a different perspective: Given the wide variety of OSNs, can the investigations on different platforms reveal a more comprehensive picture of people’s general public responses towards certain disasters? Do people behave similarly on different platforms and can we gain new insights using data collected from multiple online social platforms or channels? Although researchers consider social media as an active fertile ground for contemplation and study, these publications frequently focus on one platform only or are limited to Twitter and Facebook. Nevertheless, like Reddit, other platforms can provide diverse insights as users engage with that channel differently than Twitter, particularly during earthquakes, based on our study.

To answer these questions, using a unique earthquake sequence that occurred in Southern California (SoCal) in 2019 as a case study, we provide an empirical illustration of people’s cross-platform engagement on two leading OSNs, Twitter and Reddit, a leading microblog platform and a popular news aggregation platform^4^. While Twitter leverages a following-follower structure, Reddit centers around subreddits which are communities with different interests^5^.

The Ridgecrest earthquake sequence is relatively unique because two major earthquakes (one M6.4 foreshock and one M7.1 mainshock) hit the same area (Trona/Ridgecrest, CA) within a short period of time (July 4 to July 5). It was the first large earthquake(s) to be felt widely in Southern California in 20 years^6^. This unique event sequence provides an opportunity to study people’s responses and the potential cumulative effects of such sequential extreme events^7^. Further complicating the earthquake response online, this was the first time that ShakeAlert, the earthquake early warning system of the West Coast of the USA, would have been able to provide alerts. However, the publicly available app, developed by the City of Los Angeles, did not alert users^8^, which caused users to react in a variety of ways online.

The use of social media channels to communicate and information-seek has some theoretical basis. We note that the Uses and Gratifications theory is particularly useful for our research, as it suggests that people access specific media channels based on how this channel reflects their personal values or sense of self^9^. Massey et al.^10^ found that the Uses and Gratifications theory applies to information-seeking behavior during the 1989 Loma Prieta earthquake. Further, this theory was explored in the 2015 Nepal earthquake with Twitter^11^. These networks, such as Reddit and Twitter, can also benefit crisis response as communities of online volunteers self-organize to assist from around the world^12,13^. Uses and gratifications theory contributes to our understanding why people may choose these different channels as well as how they express emotions, share information, and converse with each other about these events.

Given the importance of social media during a crisis, we found a dearth in the literature about how people responded to the same natural hazard on different OSNs. The vast majorities of studies focus on Twitter or Facebook, but rarely multiple platforms or channels in tandem, to compare and contrast this discourse. In a recent study by McBride et al.^14^, news media and social media discourse were compared and contrasted however this is not a common method. In this work, building upon a previous study by Ruan et al.^7^, which analyzed public responses to the 2019 Ridgecrest earthquakes on Twitter, we conduct a cross-platform analysis that combines Twitter and Reddit data to explore the following questions regarding public response to the earthquakes:

**RQ1**: What were the similarities and dissimilarities between users’ responses on Twitter and Reddit?

**RQ2**: Considering the different platforms of Twitter and Reddit, what is unique about people’s responses on Reddit as compared with Twitter?

We have collected a dataset containing 510,579 earthquake-related tweets and 45,770 Reddit posts (1437 submissions and 44,333 comments) posted between July 3rd and July 10th. After careful data preprocessing procedures, we compare the topics, emotions, and temporal variations between Twitter and Reddit.

By focusing on cross-platform OSN analysis of public responses to natural hazards, our work makes the following contributions:

1. Extracted Reddit posts (including submissions and comments) that are related to the 2019 Ridgecrest earthquake sequence, along with filtering such as checking the ratio of earthquake-related comments under individual submissions;
2. Identified different responses to the same earthquakes on Reddit and Twitter. More specifically, users’ responses on Reddit were much less emotionally negative and covered more diverse topics than those on Twitter;
3. Identified the most popular subreddits discussing earthquakes during the Ridgecrest earthquake sequence, and one of the most popular venues was *r/conspiracy*, indicating that rumor discussions may be more prevalent than expected; and
4. Discovered diverse responses in different subreddits, as reflected by users’ response time and conversation networks in the main subreddits during the earthquake sequence.

## Related work

### Use of social network during extreme events

Social media has become an increasingly important tool for people’s communication and news aggregation. Twitter and Reddit are two of the leading OSNs. Researchers have been utilizing the OSNs to collect information for extreme events, including both natural hazards and social events^1–3^. Case studies include photos of the 2007 Southern California wildfire^15^, the 2010 Haiti earthquake^16^, the 2017 Hurricane Harvey^17^, the 2019 Indonesia fire^18^, and earthquake detection using Tweets^19–21^. Some closely relevant research also discussed people’s responses to natural hazards, such as earthquakes^22^, the 2012 Hurricane Sandy^23^, the 2015 Typhoon Etau^24^, the 2016 Hurricane Matthew^25^, and the recent COVID-19^26^. Most studies only focused on a single platform, and there is limited work on cross-platform analysis. However, many different platforms are becoming increasingly popular and people use them with different motivations^27^. Therefore, it is important to understand whether these studies on one single OSN can give us a full picture of people’s general responses to extreme events.

### Topic modeling and emotion analysis for short text

The most well-known technique for topic modeling is latent Dirichlet allocation (LDA)^28^ and it is effective for analyzing long documents. However, most posts are relatively short on OSNs. For example, Twitter is based on messages (i.e., tweets) with a limit of 280 characters in length, in which case LDA is not suitable due to the rare word-occurrence. Previous research has proposed new algorithms designed for short text topic modeling. The literature on short text topic modeling describes four overarching categories: Dirichlet multinomial mixture (DMM) based methods^29^, global word co-occurrence-based methods, self-aggregation based methods^30^, and pseudo-document-based topic model^31^. In our study, we performed one of the DMM based method named GPU-PDMM and used another global word co-occurrence-based methods named word network topic model (WNTM)^32^ to verify the results because they have been shown to perform well on Twitter and Reddit data^33^.

Emotion analysis can be regarded as a computational treatment of opinions, sentiments, and subjectivity of text in order to find the viewpoint of authors on specific entities^34,35^. Linguistic Inquiry and Word Count (LIWC) is a software that has been widely used for emotion analysis in social media study^36^. It evaluates the frequency of a certain corpus containing words in predefined psychological or structural categories^36,37^. We utilized LIWC to analyze the proportion of positive/negative words in tweets/ Reddit posts and explore how people’s emotions vary on these two platforms.

### Cross-platform OSN analysis

There are many different social media platforms driven by different conceptual frameworks and motivations, and they are used by different groups of people. These platforms can provide different types of information during the same disaster. As such, cross-platform OSN analysis can potentially generate useful new insights for crisis informatics from different perspectives. However, since most prior works concentrated on single-platform analyses, analyses are lacking in the social media research domain, especially in crisis informatics analysis, with cross-platform data. While study by Hall et al.^38^ gives an overview that addresses the “methodological, analytical, conceptual, and technological challenges and opportunities of cross-platform analysis in social media ecosystems”, limited cross-platform analysis exists in the literature that uses cross-platform analysis to explore public responses in crisis^39–43^.

## Methodology

### The 2019 Ridgecrest earthquakes

We focus this study on the earthquake sequence near Ridgecrest in Southern California in July 2019. A M6.4 foreshock occurred on July 4 at 10:33 a.m. PDT, and 34 h later, another M7.1 mainshock struck again on July 5 at 8:19 p.m. PDT along with more than 100,000 aftershocks^44^. Given these earthquakes (M6.4, M7.1) were felt by a large number of people, we focus our study on these two events.

### Public responses to earthquake sequence

We used Twitter and Reddit to conduct our study to investigate cross-platform public responses on OSNs during the earthquake sequence. Both Twitter and Reddit are leading OSNs used by millions of people globally but they have very different structures and mechanisms, thus people using them have different motivations^27^. Unlike Twitter, which is based on micro-blogging of information with maximum of 280 characters, Reddit has a 40,000 character limit (https://www.reddit.com/r/changelog/comments/39hf9x/) and contain subreddits, where people can congregate for certain topics. Many of these subreddits are user-created, with thousands of different groups throughout the site^45^. These subreddits bring together people by interests in specific topics, communities of practice, or geographical areas^46^. Using User and Gratifications theory as a framework, we suggest that interactions with these subreddits align with people’s sense of self and values. In contrast, Twitter allows anyone to send and receive 280-character text messages (tweets) via any Internet-enabled device, such as a Web page, mobile device, or third-party Twitter applications. Twitter does not have similar community-level information as Reddit but Twitter allows users to follow each other and therefore forms another type of “community” created by follower–followee relationships and their postings. Twitter also has many accounts with verified identities while Reddit is anonymous. The different mechanisms of two platforms can provide complementary information to characteristic public responses. In previous Twitter analysis, the verified account information was used to explore how different accounts including authorities (e.g., @USGS: U.S. Geological Survey), news media (e.g., @latimes: Los Angeles Times) and celebrities responded in the earthquake sequence^7^.

Different aspects can be used for OSN analysis, including structures, content, and user behaviors^47^, reflecting Uses and Gratifications theory. We compare the corpus on Twitter and Reddit from the following aspects: emotion, topic, and user responses. Emotion analysis and topic modeling are two effective approaches to capturing how people felt about the events and what topics attracted people’s attention. Response time is another aspect that can be used to examine how responsive the users were on different platforms to these earthquakes.

Owing to the difference between Twitter and Reddit, even though there are some conversations on Twitter, most tweets are not replied to or retweeted, while on Reddit, people’s conversations are more pervasive. Some users posted submissions while other people then discussed the post in the comments. Therefore, those conversations between users represent critically important content on Reddit. Due to the special mechanism of Reddit, we also performed the following analysis based on its unique features, e.g., subreddits and user conversations:

1. We examined diverse behavior by users on the different subreddits during the earthquake. Specifically, we examined users’ response time in the main subreddits;
2. Based on the conversations of users, we constructed earthquake-conversation networks in those subreddits. We visualized these networks and used some quantitative measurements to quantify the differences among them.

#### Twitter data collection and filtering

Our Twitter data were collected from Pushshift (https://pushshift.io), which utilized the Twitter Stream API to obtain 25,376,348 tweets from July 3 to July 10, 2019, around the epicenter of the M6.4 foreshock. Note that July 3 to July 10, 2019 is the time period for the data collection since it covers the Ridgecrest earthquake sequence, but in the later analysis, we only need to focus on a shorter time period around the event. In order to select the earthquake-related tweets, we used the following keyword list to filter the relevant tweets: *‘earthquake,’* *‘gempa,’* *‘temblor,’* *‘terremoto,’* *‘sismo’* from a previous research by Earle et al.^48^, and *‘aftershock,’* *‘epicenter,’* *‘tremor,’* *‘seismometer,’* *‘seism,’* *‘seismology,’* *‘seaquake,’* *‘epicentre,’* *‘seismicity,’* *‘ridgecrest,’* *‘ridgecrestearthquake,’* *‘quake’* as new keywords in our research.

However, some of the remaining tweets were not relevant to the earthquakes of interest. We excluded earthquakes that happened in other places through another keyword list: *‘canada,’* *‘british,’* *‘vancouver,’* *‘china’*. A soccer club named San Jose Earthquakes Soccer Club also led to many irrelevant tweets so we used *‘sjearthquake,’* and *‘quake74’* to remove them. Finally, we verified the language feature in the raw data and only kept the English tweets, which resulted in 510,579 tweets in the end , which were contributed by 314,583 unique Twitter users (1.62 Tweets/user)).

#### Reddit data collection and filtering

The Reddit data were also collected from Pushshift^49^. Reddit has a different structure from Twitter and two different datasets were provided: RS (i.e., Reddit submissions) and RC (i.e., Reddit comments). Pushshift maintains all the Reddit data in its database and releases monthly Reddit data. We, therefore, used the RS and RC for July 2019. Unlike the Twitter data, in which we limited tweets geographically around the epicenter, the Reddit data were from the whole platform and therefore included much more irrelevant data. We performed more complicated filtering to further refine the earthquake-related data.

**Figure 1:**
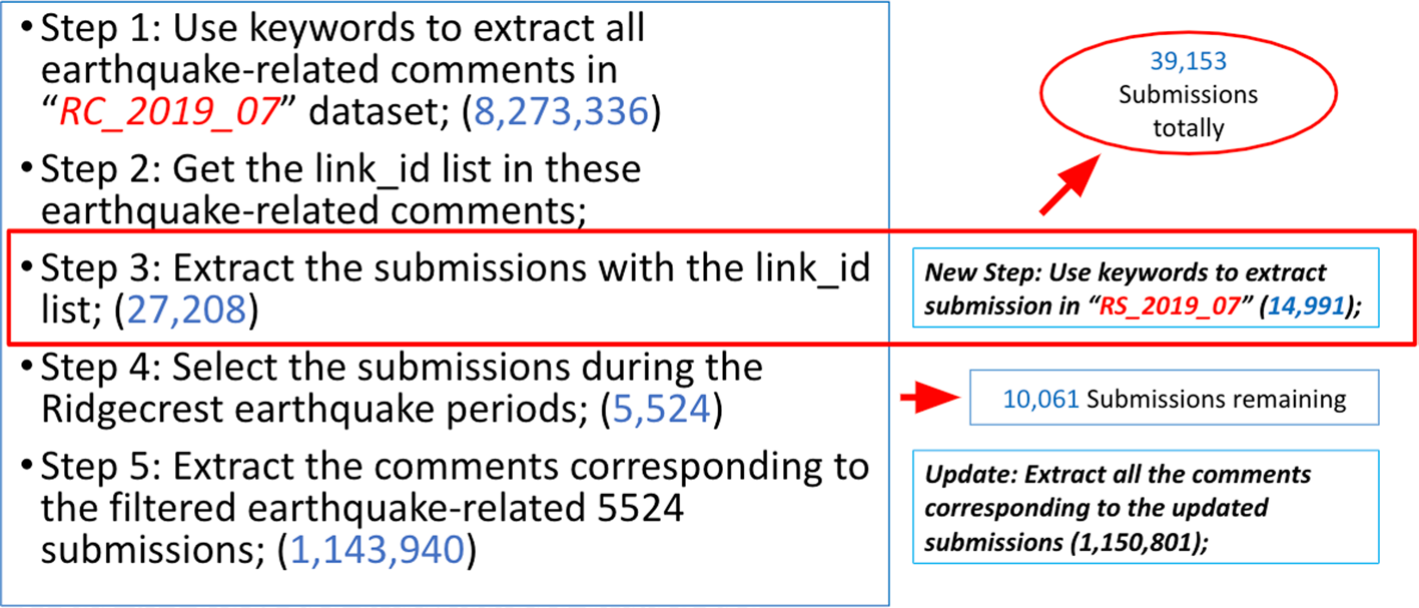
Filtering earthquake-related posts from the Reddit RC/RS datasets.

Figure 1 elaborates our preliminary data filtering process. Because the Reddit raw dataset is stored on a monthly basis, we need to start from the whole July 2019 dataset. First, we traversed the *RC_2019_07* (Reddit Comments in July 2019) dataset and used the same keyword listed above to obtain all the earthquake-related comments (about 8 million). Then based on the *‘link_id’* feature of those comments, we retrieved 27,208 corresponding submissions. Meanwhile, we also used the same keyword list to directly check the *RS_2019_07* (Reddit Submissions in July 2019) dataset and extracted 14,991 submissions. The two sets of submissions (39,153 in total due to some overlap) and their comments constituted our preliminary earthquake-related Reddit posts.

**Figure 2:**
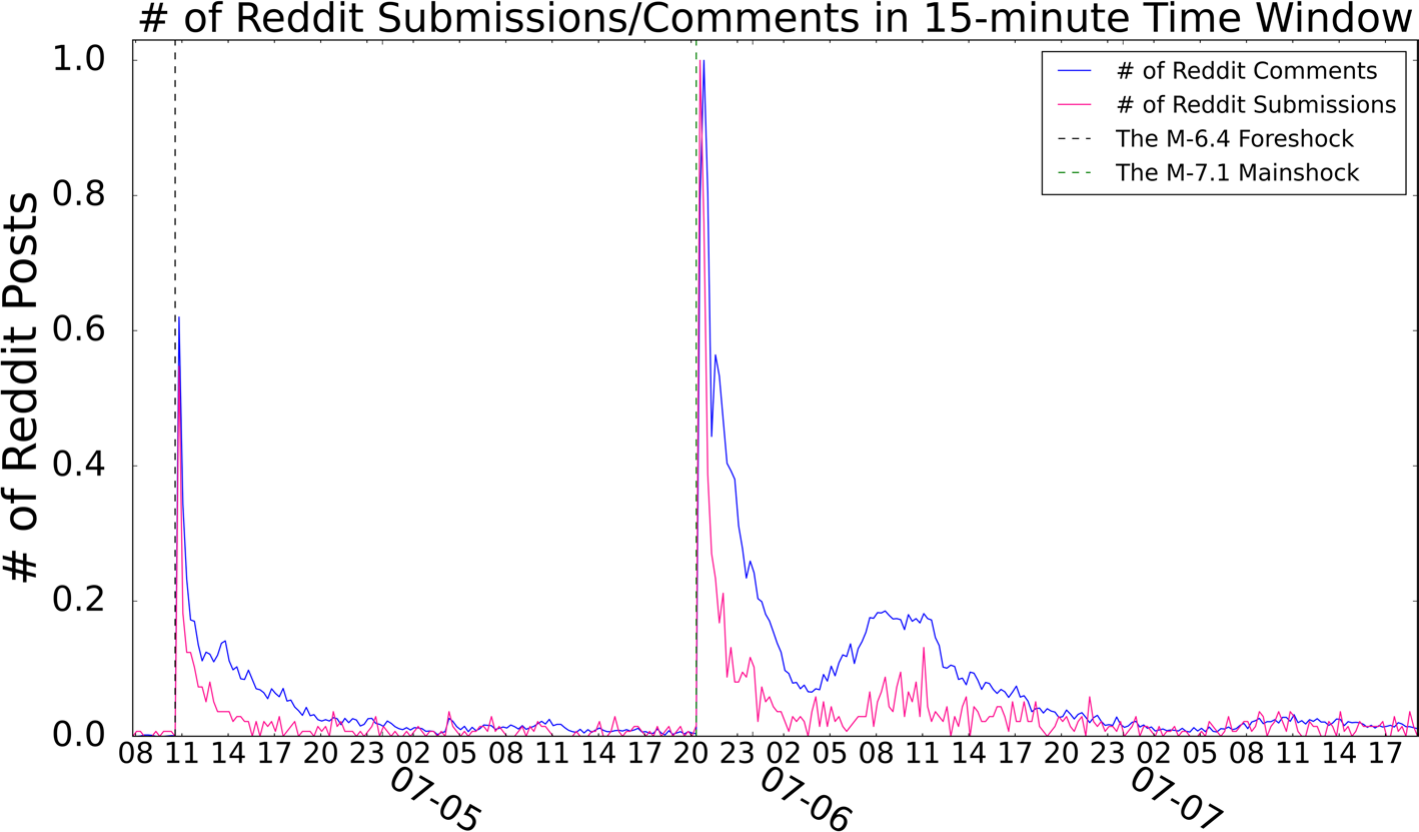
Number of earthquake-related posts from the Reddit RC/RS datasets (the numbers are normalized, i.e. 1.0 means the peak number of earthquake-related posts in RC/RS). 1437 submissions and 44,333 comments are included in the plot.

However, this preliminary collection still contains “noisy” data. We discovered that some comments were related to earthquakes but most of the other comments were not. For example, we found some popular sport game threads during our study period had a number of related comments but few users mentioning the actual earthquakes. In order to exclude such cases, we further filtered the Reddit posts. For the first set of submissions (from comments’ *‘link_id’*), we checked their comments. The submissions were retained only when the ratio of comments containing earthquake-related keywords was larger than 15% and the number of such comments is larger than 5. For the second set of submissions (from directly checking *RS_2019_07*), when the earthquake-related comment ratio is more than 15%, the submissions were kept. The second submission set used a looser standard because we found submissions were much more likely to be related to the earthquake topic if the submission body included earthquake-related keywords. Following this method, we collected 45,770 Reddit posts (including 1437 submissions and 44,333 comments), which were contributed by 25,462 unique Reddit users (1.79 posts/user), for Reddit analysis. Figure 2 shows the number of Reddit submissions and comments in a 15-min time window after our filtering process. Similar to the findings in^7^, two peaks of activity started shortly after the actual occurrence of the two major earthquakes, which verifies the rationality of our filtered Reddit data. Besides 15-min time window, we also examine other time windows including 5-min, 10-min and 30-min. All different time windows present consistent results, and we pick 15-min here because the result is smooth and also representative.

### Preprocessing

Before applying topic modeling on the tweets or Reddit posts, preprocessing was required. In our study, we used standard natural language processing methods to preprocess all the corpus. All the mentions(@), hashtags(#), punctuation, and URLs were removed through regular expressions. We also used the *simple_preprocess* function provided in the *gensim* python package to strip tags, punctuation, multiple white-spaces, short words, and digits as well as remove stop words. All sentences were lower-cased, tokenized, and de-accented so that a list of tokens were obtained for each tweet or Reddit post and they were prepared for topic modeling. Finally, we removed all non-English tokens using the English dictionary.

### Time division

In order to compare people’s responses to the two earthquakes on the OSNs, we used time windows. The first time window is between the foreshock and mainshock, while the second one is from the mainshock and with the same length as the first one. These two windows of the same length can help us directly compare the time periods.

## Comparison of Twitter and Reddit data

In this section, we aim to answer RQ1: What were the similarities and dissimilarities of the earthquake-related public responses between Twitter and Reddit?

### Emotion analysis

We used LIWC (Linguistic Inquiry and Word Count)^36^, which has been introduced in the “Related work” section, to detect the proportion of emotional (positive vs. negative scores) language used in the different corpora. LIWC is a popular software widely used in social media research that counts words in psychologically meaningful categories. Two corpora are constructed from the tweets and Reddit data before and after the mainshock separately, which represent people’s responses after the foreshock and after the mainshock on the two different OSNs, respectively.

**Figure 3:**
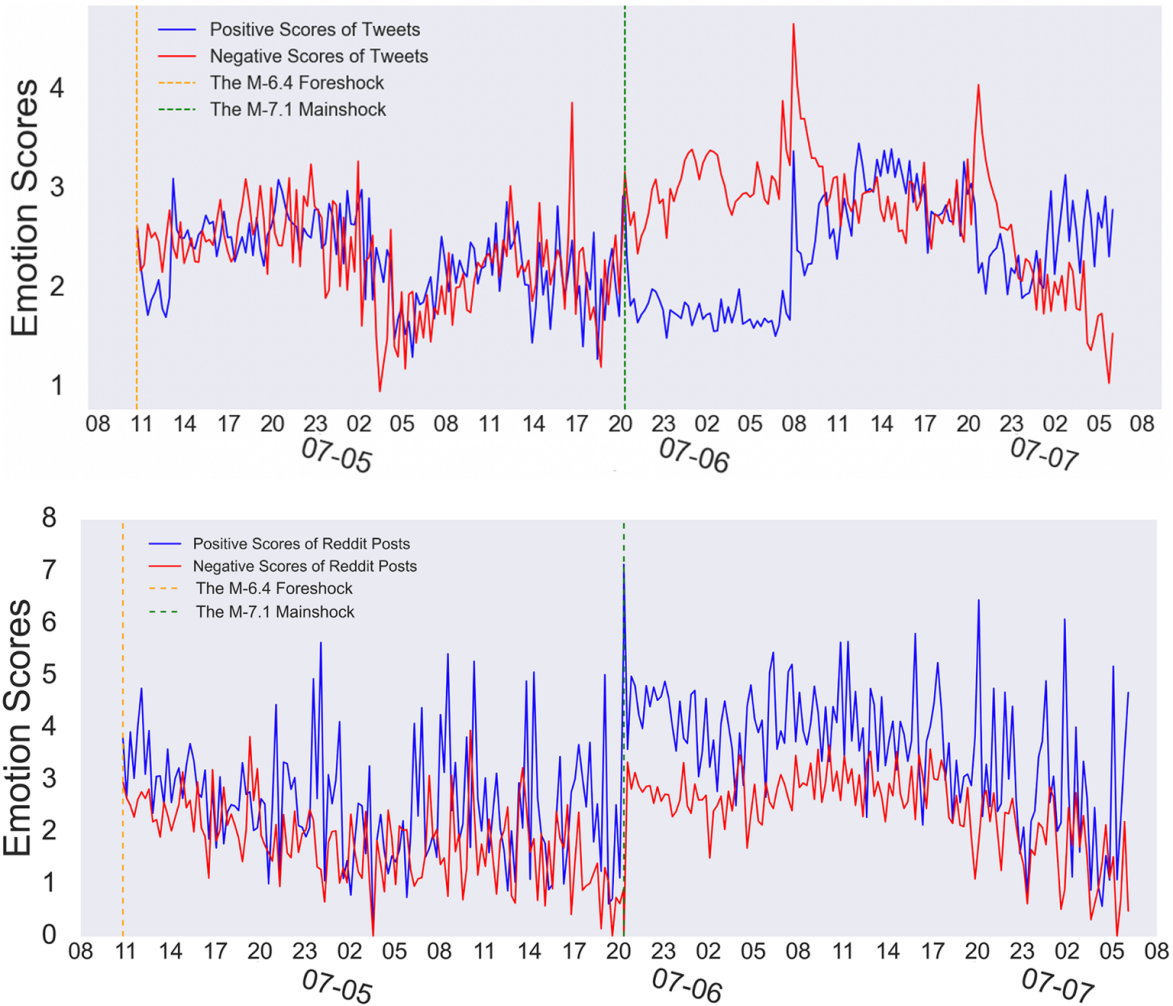
Emotion scores of people’s tweets and Reddit posts in every 15-min time window. The emotion scores in this plot are calculated based on the percentage of positive and negative emotion words within a text string, e.g., a positive score of 8 here means that on average, 8 percent of words in the documents during the 15 min are positive words.

We plot the time series of the mean negative/positive LIWC scores in every 15-min time window in Fig. 3 to illustrate the temporal difference of people’s emotions after the two earthquakes on Twitter and Reddit.

#### Emotion differences

Figure 3 plots people’s emotional dynamics during the earthquakes. We observe significant differences in emotions on the two OSNs. As discussed in the previous study by Ruan et al.^7^, Twitter users became increasingly anxious after the mainshock, shown by the immediate large deviation of positive and negative LIWC scores after the mainshock. People’s overall negative emotion was larger than positive emotions. However, Reddit’s patterns scored higher positively than negatively. These positive scores may indicate that users tend to express their negative feelings on Twitter, possibly due to the word limit restrictions on Twitter. Twitter is also used widely for the ongoing discussion and instant evaluation of newsworthy events^50^. Therefore, people may have rushed to Twitter and expressed their immediate feelings shortly after the event. In contrast, Reddit does not have a length limit and is more like a news aggregation platform. People have more time to discuss and reflect on events, rather than express their immediate emotions about the earthquakes.

### Topic modeling

As discussed in the “Related work” section, GPU-PDMM was employed in our study to compare people’s response topics after the major earthquakes on two different OSNs. More specifically, we applied GPU-PDMM on each corpus after the foreshock and mainshock respectively. The output keywords by GPU-PDMM can help identify people’s focuses after the two earthquakes on different platforms. In addition, WNTM was applied to compare with GPU-PDMM results. There was considerable overlap between the topics output from the two topic modeling approaches (67% overlapping topics for foreshock, 80% overlapping topics for mainshock). We used the GPU-PDMM results for further analysis since GPU-PDMM utilizes the semantics of words (word-embedding) and its modeling results tend to have better cohesion.

We explored different topic numbers in the application (5, 10, 15, etc.) and manually compared the quality of the topics identified. We found that using 15 topics yields the most explicit and comprehensive topics. Meanwhile, we utilized coherence score for assessing the quality of the learned topics and 15 topics output a reasonable topic coherence.

#### Topic modeling results

Tables 1 and 2 show the output keywords of topics on Reddit by GPU-PDMM for the foreshock and mainshock. Comparing the results with the topics on Twitter^7^, Reddit topics have obviously different features from those from Twitter. As discussed in the previous Twitter analysis work, emotional keywords can be observed in many topics and thus emotional (either positive or negative) topics were common both after two earthquakes on Twitter. However, Tables 1 and 2 contain fewer emotional keywords. Instead of expressing personal emotions, Reddit posts are descriptions, covering a vast range of topics. We also observed note-worthy topics on Reddit that never appeared on Twitter. For instance, the ***Alert*** topic clearly indicates that people were discussing the earthquake early warning and performance expectations. As discussed in the “Methodology” section, during our preprocessing steps we followed the standard approach to remove all the words that are not in the English dictionary, and “ShakeAlert” was therefore removed. That is why “ShakeAlert” is not in the list of keywords. To the best of our knowledge, ShakeAlertLA was the only EEW app that could send alerts to the Los Angeles region at that time. It is possible that some of the people were talking about other earthquake notification apps but the topic modeling process combined them into the same topic.

The ***Nuclear*** topic focused on one rumor that the earthquake was due to a nuclear bomb test in the Naval Air Weapons Station (NAWS) at China Lake near the epicenter. The ***Hazards*** topic indicates that Reddit users also discussed other natural hazards (e.g., hurricane, tornado) when the earthquakes happened.

There were a few topics that crossed platforms. For example, the ***Big one*** has been shown to be a popular rumor topic on Twitter^7^ (Big one means an extremely large earthquake of M7.8 or even higher striking California) and people were also actively talking about it on Reddit. The ***Self-rescue*** and ***Preparedness*** are two other common topics on both platforms that focus on how people should protect themselves (e.g., whether people should run or hide and where is the safest place indoor) and what should be prepared (e.g., food, water, toolkit, etc.) during natural hazards. These topics generated interest and discussions from users on both platforms which may represent more general public concerns after the large, impactful earthquakes. It is notable that “Self-rescue” topic appears on Reddit both after foreshock and mainshock. Some of the keywords in this topic are very relevant to damage description such as “fall, building, shake”. We conclude the topic is about “Self-rescue ”due to the fact that when people talked about keywords like r“un, outside, stay, cover, safe, head” in Table 2, they were always discussing what is the correct choice during an earthquake: to run outside or stay inside and find someplace to cover the head. We assign the “Self-rescue” topic in Table 1 because this topic shares many keywords with the “Self-rescue” topic in Table 2 but we acknowledge that the “Self-rescue” in Table 1 is not as obvious as “Self-rescue” in Table 2.

The topic modeling results and the comparisons between Reddit and Twitter show that a single platform analysis can only partially cover the general public responses. When combining two or more OSNs we can potentially obtain a better comprehensive understanding of the public responses. This can assist science and emergency management agencies to discover and address issues of public interest more effectively. We can use the “Southern CA” topic after the foreshock as an example: this topic is an example of people reporting the specific locations that were severely affected by the earthquake. Typically such information needs to be collected by surveys (e.g., “Did You Feel It?” by USGS Earthquake Hazards Program). With the OSN data, we can obtain this information in a different way. Emergency management agencies can potentially get such kind of actionable information more promptly and allocate the rescue resources to the regions that need help most.

**Table 1:** Top 15 topics on Reddit after the M6.4 foreshock using GPU-PDMM. Topics on Twitter can be found in^7^.

Topic type	Keywords
Description	Earthquake fault get go area would quake place time people stress see much really say give event lot good shake
***Self-rescue***	Fall get building thing shake run build work wall house hope good head safe break shelf floor old table damage
Description	Feel think go shake start bed move back first thing still sleep see wake realize look sway car sit whole
Description	Move right state sound road snow run ground side high cool see cause water earth look get mountain place entire
Description	Feel shake second long pretty roll last good get strong wave minute bit way definitely little sway floor check slow
Insurance	Water fire go house emergency home gas power store stuff line keep need damage buy use insurance open lot work
***Negative***	Get go live say think see still look guy thing right would feel back fuck die love work family friend
Forum	Post question comment thank use look ask link bot action concern report try help source rule moderator remove new find
Description	Earthquake big time happen quake year live really know first even people say one never day also much make ever
NBA	Play well amp game good would go start think take use probably money team make none time late give card
Southern CA	Quake area say big damage aftershock magnitude also epicenter time far large report hit mile people small southern chance
***Negative***	Earthquake people know make even see shit take watch happen say fuck post let news movie sure probably really seem
***Alert***	Would want alert app work may way give could point set warn send use put get actually shake maybe location
***Nuclear***	Fault would could large think magnitude see test energy likely scale major plate look weapon release nuclear show event cause
Description	Good place food go get find way live well great pretty new thing still taco much city try eat bad

**Table 2:** Top 15 topics on Reddit after the M7.1 mainshock using GPU-PDMM. Topics on Twitter can be found in^7^

Topic type	Keywords
Description	Earthquake go get quake time make would people know fault big even thing may say year building could think feel
***Nuclear***	Cause earth base earthquake test evidence say weapon natural ground call fact deep military great mile human bomb science underground
Insurance	Building insurance home cost pay build need state money regulation high city structure price retrofit house code require old new
Discussion	Fault large magnitude scale chance aftershock likely occur would event cause different wave zone high plate move energy predict release
Rescue	Use work call would go stop alert amp get train surgery system patient hospital second need want send take emergency
***Big one***	Earthquake big quake happen live time year feel one say know hit even day really pretty area come much also
***Preparedness***	Water need food case put bag make emergency supply power pool buy good work keep kit gas store plan eat
***Negative***	Get go think people shit see fuck right take make back bad look would want dog way scary hear hope
***Hazards***	People get damage would go fire tornado bad hurricane take cause city think well see way least probably major kill
Description	Feel shake second move start long yesterday sway first back minute definitely bit aftershock little roll ground rock sit pretty
Description	Earthquake know say really time happen could even also still much live may come quake sure tell actually place lot
Forum	Post comment question thank link use read find report edit bot action ask see concern remove moderator make try message
Unknown	Go would think make good get people thing see way well bad probably look take hope want right try always
***Self-rescue***	Fall building shake build table run house collapse move stay outside safe wall desk thing bed cover head doorway floor
NBA	Look video see game watch play think make fake would go get movie people right end show room keep stop

### Response time

To explore how people responded differently on the two OSNs, we use response time as a measure of response efficiency to compare them, which is defined as the time duration between the original post and retweets/comments of that post^51^.

Furthermore, we explored how users on those two platforms responded differently to the external information, such as the URLs pointing to other websites. We first extracted all the external URLs cited on the two OSNs and then obtained the common 197 URLs that appeared on both platforms.

**Figure 4:**
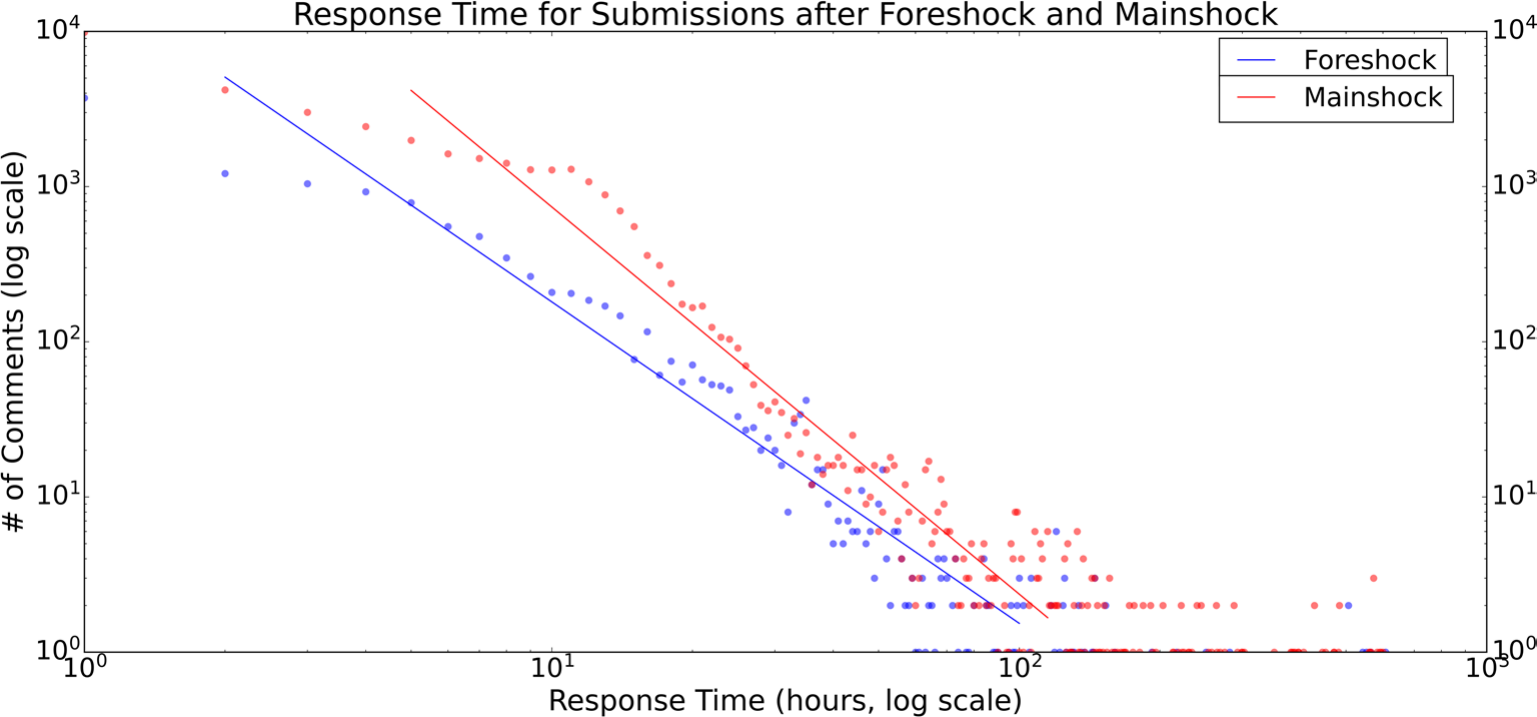
Power-law in response time on Reddit. Response time on Twitter can be found in the paper by Ruan et al.^7^.

Similar to what has been reported for Twitter users^7^, people’s response time on Reddit also followed the power-law phenomenon but had an obvious flat area as shown in Fig. 4. The reason for the flatter slope is due to the different mechanism that Reddit has: Reddit users may have posted the submissions in the evening and many other people replied to them the next morning. This was a common scenario on Reddit but not on Twitter.

**Figure 5:**
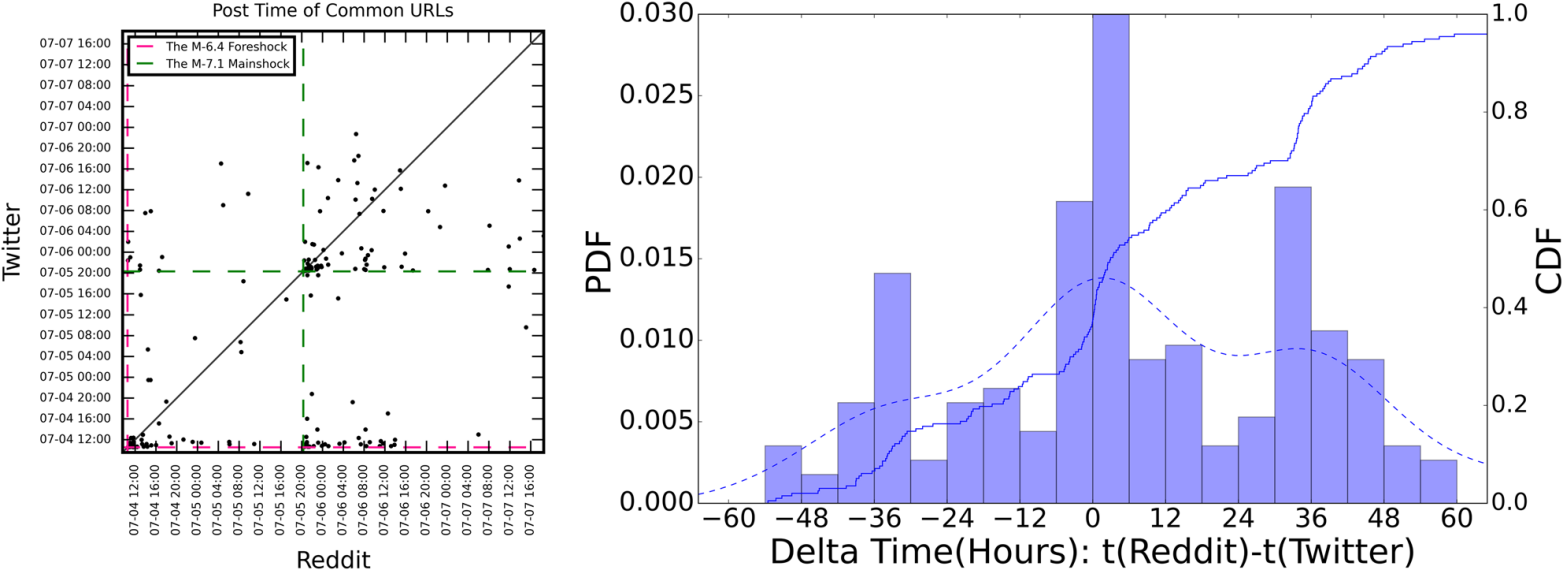
Common URLs’ post time and response time difference on two OSNs. The dashed line and solid line in the right plot correspond to the PDF curve and CDF curve of response time difference, respectively.

Figure 5 plots the CDF of the response time difference for the common external URLs and the scatter plot for the posted time for the common URLs on two OSNs. As shown in the scatter plot, for the common URLs, Twitter users had much faster responses towards the external information than Reddit users since more points are below the y=x line. More specifically, 127 of the 197 common URLs were cited on Twitter first while only 70 appeared on Reddit first. The CDF plot also supports this observation: t(Reddit) - t(Twitter) $\in$ [0, 6 h] has the highest bar, which indicates more common URLs appeared 0–6 h earlier on Twitter than Reddit. It is noteworthy that there are two peaks at around ± 30 h. This is because 34-h is the time between the foreshock and mainshock. Some common URLs were first cited on one platform after the foreshock and then repeated on the other platform after the mainshock.

## Unique information retrieved from Reddit

Our second study aims to answer RQ2: Considering the different mechanisms between Twitter and Reddit, what unique information and insights about public responses can be gained from Reddit?

### Subreddit analysis

To explore which subreddits were the most popular places where Reddit users talked about the Ridgecrest earthquakes, we examined each post’s subreddit and sort the subreddits by the number of posts. We found many different subreddits got involved in the Ridgecrest earthquake discussions, among which the most popular subreddits were r/news (12,702), r/LosAngeles (3864), r/conspiracy (2414), and r/Earthquakes (1942). Those subreddits’ names represented different themes of the user groups: r/news is where the latest news is aggregated and discussed while r/LosAngeles is the nearest large metropolitan area where people felt light to moderate shaking, and r/Earthquakes focuses specifically on earthquakes. Note that r/conspiracy was also one of the most popular subreddits. According to previous research, many earthquake-related rumors indeed spread in California locally^52^. It is intriguing that the conspiracy became people’s main focus on Reddit, which has users from around the world and is not subject to geographic constraints.

#### Response time within subreddits

**Figure 6:**
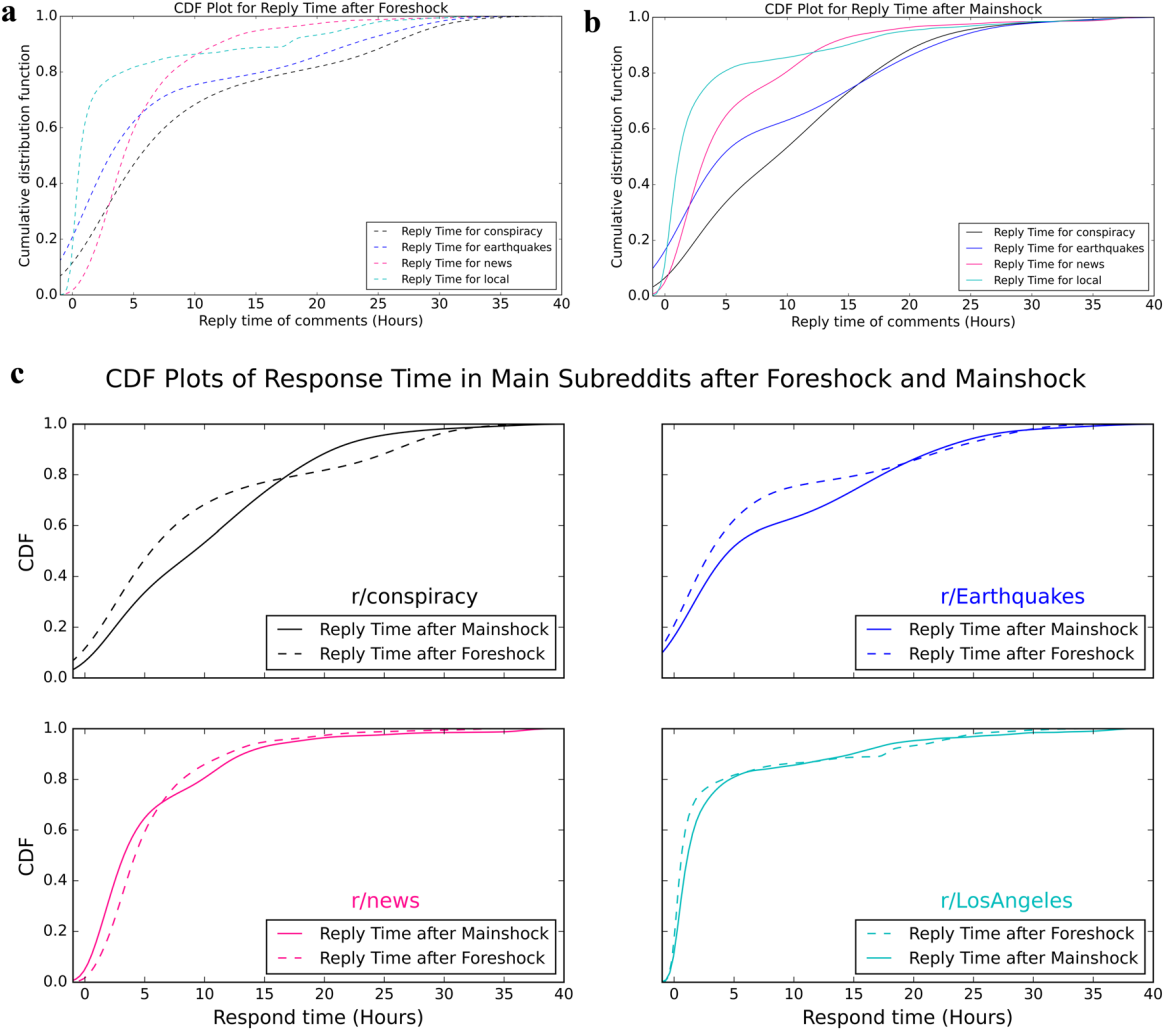
Common URLs’ post time and response time difference on two OSNs.

We examined how users in different subreddits behaved during the earthquakes. Specifically, we looked into users’ response time in these four main subreddits. Figure 6 shows the CDF of how users’ response time differs in these four subreddits.

The first two plots in Fig. 6 indicate that after the two earthquakes, the local subreddit (r/LosAngeles) and news subreddit were the quickest to respond, while the r/Earthquakes and r/conspiracy were slower. This is reasonable since, after the earthquakes, people first turned to the geographic subreddit, to gain local perspectives and news subreddits to information seeking from media outlets. Then people search or post more detailed information on r/Earthquakes; we suggest this may be because the events seemed less pressing. The conspiracies were attractive to users but our research suggests it requires more time to create conspiratorial associations or stories.

The bottom 4-panel plot in Fig. 6 intends to compare how users on the same subreddit responded differently after foreshock and mainshock. Notably, people responded faster to the foreshock than the mainshock in the three of the subreddits (r/conspiracy, r/Earthquakes, and r/LosAngeles). It may seem surprising considering the mainshock was much larger than the foreshock. One potential reason for this could be because the mainshock occurred in the evening thus most discussions occurred the next day. However, the r/news subreddit was different: timeliness is more important on r/news than others and users in this subreddit typically paid attention to recent news with fewer people would respond to news from the previous day.

#### Conversation networks

Based on the conversations between users, we constructed the conversation networks in the four popular subreddits.

**Figure 7:**
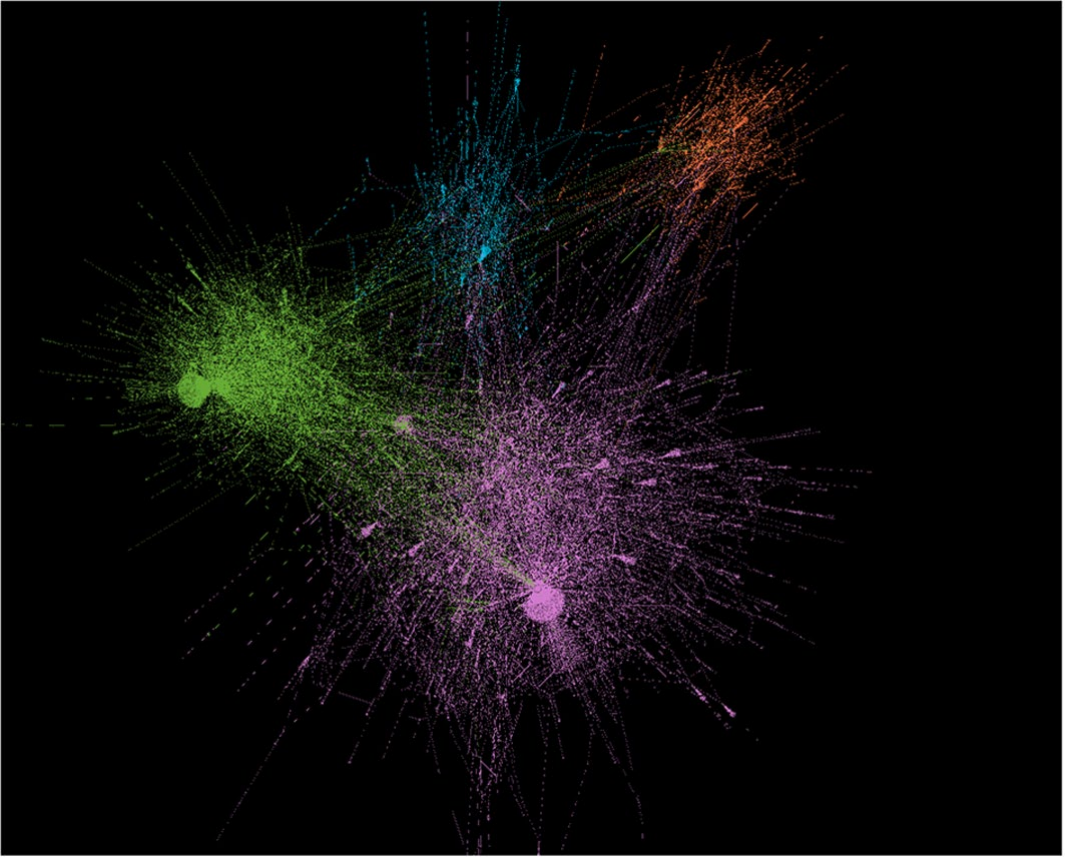
Conversation networks generated in Gephi^53^ v.0.9.2 (https://gephi.org/). Note that this figure is a descriptive visualization of the interactions among the major subreddits where discussions happened. We can see many users were active across different subreddits. Also, some subreddits such as r/news and r/LosAngeles have “central” users who attracted much attention from others. The distance in the figure does not indicate any measurement of distance between subreddits. Magenta: r/news, Green: r/LosAngeles, Orange: r/conspiracy, Cyan: r/Earthquakes.

To visualize the networks, we used Gephi^53^ to obtain Fig. 7 which shows how users in the four subreddits had conversations with others during the earthquakes. The subreddits’ interaction patterns were also different even though they were talking about the same event.

To quantify their differences, we calculated some features for the four networks. We did not use measures such as diameter and density due to the different sizes of the subreddits. Instead, we utilized measures that are agnostic to network sizes, such as transitivity and reciprocity^54^.

**Table 3:** Conversation network measures in different subreddits. Reciprocity is the likelihood of mutual connections. Transitivity is the probability that the adjacent nodes of a node are connected. The columns Mean, SD, Median, Min, Max are calculated for degrees. Note that reciprocity and transitivity calculations are based on the whole graph instead of each node so standard deviations cannot be derived as degrees.

	# of nodes	# of edges	Reciprocity	Transitivity	Mean	SD	Median	Min	Max
r/conspiracy	626	1141	0.368	0.0457	3.64	6.44	2	1	82
r/Earthquakes	610	971	0.315	0.0277	3.18	6.44	2	1	125
r/news	4900	7390	0.241	0.0266	3.01	20.4	1	1	1362
r/LosAngeles	2486	4386	0.209	0.0652	3.53	25.3	2	1	1240

As illustrated in Table 3, we used degree (i.e., how many interactions with others per user), reciprocity (i.e., the likelihood of mutual connections), transitivity (i.e., the clustering coefficient, or the probability that the adjacent nodes of a node are connected) to compare different subreddits’ structures. From this table, we can see that r/conspiracy has high transitivity (indicating more clustered around a few nodes) and the highest reciprocity (indicating more mutual conversations); Subreddit r/news has very low transitivity (connections are relatively evenly spread among all the nodes), and very low reciprocity (evidence of hierarchical relationships-for instance, media contributing content but unlikely to interact much)^55^. These different measures indicate that people turned to different subreddits for different purposes. As such, their interaction patterns can differ considerably across subreddits.

## Summary and impacts

Our work presents a first-of-its-kind cross-platform analysis of public responses to the 2019 Ridgecrest earthquake sequence on two different social media platforms: Twitter and Reddit. We conclude the paper with a summary and its potential impacts.

### Summary

In this work, we utilized the Reddit and Twitter data to analyze people’s responses across different social media platforms in response to the 2019 Ridgecrest earthquakes. We collected user responses from the two platforms related to the Ridgecrest earthquakes, which comprises 510,579 tweets and 45,770 Reddit posts (including 1437 submissions and 44,333 comments). When filtering earthquake-related Reddit posts, we combine keywords and the ratio of earthquake-related comments under submissions, which led to a more reasonable Reddit dataset related to the Ridgecrest earthquakes. Based on the refined datasets, we compare people’s behaviors on the two OSNs from different perspectives. We first compared users’ emotions during the Ridgecrest earthquakes on Reddit and Twitter. Our results suggest that Twitter users had communicated more negative emotions than Reddit users, especially after the mainshock. We also explored the topics discussed on the two OSNs. Topic modeling results supported the above emotion analysis results in that the Twitter corpus topics generated significantly more emotional keywords than that from Reddit while the Reddit corpus covered more diverse topics (e.g., ***Nuclear***, ***Alert***). We also examined the common external URLs on the two OSNs and explored whether Reddit and Twitter had different response patterns toward this external information. The results showed the responses to the external URLs on Twitter were more active and faster. Meanwhile, based on Reddit’s unique mechanisms, we discussed the different response patterns in the popular subreddits and explored the users’ conversations in those subreddits. We found that even on the same Reddit platform, people’s response patterns and behaviors can vary significantly, based on which subreddit they chose.

### Impacts

Aggregated responses, which are then used to develop themes, can assist emergency managers and science agencies responsible for communicating with the public. By thematically analyzing and grouping major questions or points of concern, emergency managers’ communication can be more effective in times of crisis^56,57^. This tactic was used by emergency managers during the M6.2 Christchurch earthquake response^58^, the 2008 Wenchuan and 2013 Ya’an earthquakes^59^, the 2019 Albania Earthquake^60^, and by science agencies in the 2016 Kaikoura Earthquake^61^, among other examples. These types of analysis can assist agencies to launch more effective and targeted crisis communication responses, either via using the same social media channels (e.g., Twitter or Reddit), but we argue that given the value of the insights, use the questions to also frame media responses. By not listening to social media, opportunities to engage and answer questions online may be lost, as what occurred during the Bombay Beach Swarm in 2016^14^.

Through the combination of different OSNs and performing cross-platform analysis, we can potentially help science response and emergency management agencies to gain a more comprehensive understanding of people’s concerns and public awareness during extreme events. For instance, misinformation or conspiracies can spread after natural hazards, but different kinds of misinformation may exist on different platforms. In our study case, Twitter users were actively talking about “Big one is coming” while Reddit users were talking about the earthquakes being caused by a nuclear test. Those topics can help science agencies monitor what types of misinformation are being spread online and then take corresponding actions to correct them and therefore prevent them from misleading more people. However, we found little evidence of cross-platform social media analysis in previous research, even less can be found for crisis informatics during natural hazards. Our research can be regarded as initial steps encouraging the use of more diverse data sources for exploring social aspects of disaster resilience^25^. Our work analyzes two different platforms during the 2019 Ridgecrest earthquake sequence that was felt by a large number of people and demonstrates that a single-platform analysis cannot fully represent general public responses, thus motivating more cross-platform analysis in the future to obtain a more comprehensive view. Furthermore, since these OSNs have different mechanisms, diverse methods need to be applied when extracting useful information from them.

In our research, we present a workflow of extracting useful information with different approaches on Reddit than the previous work on Twitter^7^, including filtering earthquake-related posts and performing specific analyses based on the unique structure of the Reddit platform (e.g., subreddit, conversations). Our methodology can be beneficial to Reddit analysis on other topics as well. Reddit has been largely overlooked as a platform for study, as opposed to Twitter, which has a voluminous body of research. Our results show that the combination of multiple OSNs, rather than a single platform, can help emergency managers and science response agencies obtain a more comprehensive understanding of public responses, which plays a prominent part in evaluating and enhancing collective actions for rapid reconnaissance, disaster preparedness, and recovery strategies^17^. Finally, our work is consistent with the Uses and Gratifications theory’s main argument: that users are attracted and use platforms that best reflect their values and perceptions of self^9^.

## Limitations and future work

There are several limitations in this study that could be addressed in future work. First, our study is representative of the English-speaking population and people having some experiences with earthquakes (i.e., those living in California). However, there are other languages spoken in the United States, e.g., by the Spanish communities in California. Meanwhile, US citizens on the east coast who rarely experience earthquakes can respond differently than people living in California. Public responses to earthquakes in other non-English-speaking communities and people with less earthquake experience could be explored in future work. Second, OSN users do not represent all age-groups. Based on previous research, some platforms such as Reddit are mainly used by young people (18–29). Therefore, analysis performed on those platforms may only represent the responses of younger population. Third, different cultures can prefer different OSNs, for example, Twitter is heavily used in Indonesia, but less widely used in China and Russia^62^. Therefore, even though the techniques in this study can be applied with a change of keywords and language analysis for other events, researchers should also be aware of the relevant platforms for that region. Last, our study does not address the potential change of topic trending in extreme events, partially due to the short period of our analysis. However, other extreme events such as a hurricane can last for a longer time and affect larger areas. Hence, the topics can change markedly. In this case, keywords filtering will need to be adjusted based on current events and region of interest accordingly.

## Data Availability

Our Reddit data are from Pushshift website (https://pushshift.io) and the Twitter data are collected from Twitter Academic API. Earthquake-related data can be easily retrieved following the extraction methodology of the paper. The other data that support the results of this study are available from the corresponding author upon reasonable request.
